# Reef foraminifera as bioindicators of coral reef health in southern South China Sea

**DOI:** 10.1038/s41598-021-88404-3

**Published:** 2021-04-26

**Authors:** Aishah Norashikin Abdul A’ziz, Fatin Izzati Minhat, Hui-Juan Pan, Hasrizal Shaari, Wan Nurzalia Wan Saelan, Nazihah Azmi, Omar Abdul Rahman Abdul Manaf, Md Nizam Ismail

**Affiliations:** 1 Paleoceaonography Research Interest Group (PoRIG), Faculty of Science and Marine Environment, Universiti Malaysia Terengganu, 21030 Kuala Nerus, Terengganu, Malaysia; 2 Institute of Oceanography and Environment, Universiti Malaysia Terengganu, 21030 Kuala Nerus, Terengganu, Malaysia; 3 Institute of Earth Sciences, College of Ocean Science and Resource, National Taiwan Ocean University, Keelung, Taiwan; 4 Fisheries Research Institute, 11960 Batu Maung, Pulau Pinang, Malaysia

**Keywords:** Marine biology, Ecology, Environmental sciences

## Abstract

Pulau Tioman is a famous tourist island off Peninsular Malaysia with beautiful coral reefs. This study aims to assess the health of the coral reefs surrounding Pulau Tioman based on the application of the Foraminifera in Reef Assessment and Monitoring Index (FI). Ten sampling sites around Pulau Tioman were studied with a total of 30 samples. Eight orders, 41 families, 80 genera, and 161 species of benthic foraminifera were identified. The agglutinated type of foraminifera constituted 2–8% of the total assemblages. Calcareous hyaline and porcelaneous groups represented 79% and 19% of the total assemblages, respectively. Symbiont-bearing taxa were the most common foraminifera. The results indicate that most of the sampling sites are conducive for coral reef growth with good recoverability from future stress to the ecosystem. However, several areas with higher coastal development and tourism have reduced water and sediment quality. Therefore, the limit on the number of visitors and tourists should be revised to enable coral growth and health. The FI values in this study showed a positive correlation with good water qualities and a negative correlation with organic matter enrichment. The FI is a good measure to assess the health of a coral reef and can be applied to other reef ecosystems around Malaysia.

## Introduction

The coral reef ecosystem is among the most biologically diverse ecosystems in the world that plays a vital role in shaping the balance of environmental processes over the past 200 million years^1^. Coral reefs provide goods and services to marine tropical and subtropical regions^2^. Globally, coral reef ecosystems are threatened or have disappeared due to climate change and human intervention^3^. On a larger scale, elevated sea temperatures and ocean acidification caused by climate change have challenged the natural resilience of tropical reefs^4^. Local stressors from human activities, such as coastal development and pollution, reef predatory practices, and diseases, usually reduce the potential of reef recovery from the ill effects of climate change^4–6^. Because coral reef ecosystems provide various natural environmental services, their ability to survive the global climate anomaly is important. Consequently, understanding the health status of local individual reefs is important to ensure that the coral reef survives and recovers from likely mass mortality events^4^.

Benthic foraminifera can successfully be used to monitor the potential recovery of the reef ecosystem^7–9^. Benthic foraminifera have been proven to be excellent indicators of sediment quality, heavy metal pollution, organic pollution, and water quality^10–13^. Many taxa of Unicellular foraminifera are abundant in reef ecosystems; some of these taxa have similar ecological requirements as those of reef building corals^11^. The development of the Foraminifera in Reef Assessment and Monitoring (FORAM) Index (FI) by Hallock et al.^11^ has enabled continuous monitoring of the health of coral reefs. The FI was developed as a low-cost monitoring tool to indicate whether the quality of water surrounding a reef ecosystem can support reef growth^11^. This index was first applied in the Western Atlantic reef^11^ and has subsequently been applied widely in other regions, including the Great Barrier Reef in Australia^7,8^, reefs in Brazilian waters^14^, and the Saronikos Gulf, Greece^15^. The reliability, simplicity, and cost effectiveness of the FI has made it a suitable indicator for the monitoring of coral reef.

Monitoring the environmental health of the surrounding reef is very important for natural park management authorities to safeguard the coral ecosystems and maintain their ecological importance in Pulau Tioman. This study aims to assess and monitor the health of the reef environment surrounding the Pulau Tioman island based on the application of the FI.

## Results

### Foraminiferal assemblages, functional groups, and FORAM index

A total of 8 orders, 41 families, 80 genera, and 161 species of benthic foraminifera were identified around Pulau Tioman. The most dominant species was *Amphistegina lessonii* (average of 22%) and the least dominant species (< 4%) were *Bolivina vadescens*, *Elphidium neosimplex*, *Heterolepa dutemplei*, *Heterolepa subhaidingerii*, *Mikrobelodontos bradyi*, *Milliolinella suborbicularis*, *Operculina discoidalis*, *Parahourinoides fragillissimus*, *Quinqueloculina incisa*, *Quinqueloculina sulcata*, *Triloculinella bertheliniana,* and *Triloculinella parisa*. Overall, the agglutinated foraminifera contributed 2–8% of the total foraminifera assemblages in Pulau Tioman. Calcareous hyaline and calcareous porcelaneous groups represent on average 79% and 19% of the total assemblages, respectively (Table 1). A majority of the species that made up the calcareous hyaline group were larger benthic foraminifera from the Amphistegenidae, Calcarinidae, and Nummulitidae families. As for the porcelaneous species, Miliolidae is the most commonly recorded family. The highest number of species was recorded at station J3 (S = 25) while the lowest number of species (S = 12) was at station F3. The Shannon–Wiener (H′) diversity index values around Pulau Tioman were 1.8–3.0. The species evenness (J′) values were 0.37–0.79.

**Table 1:** Benthic foraminifera distribution in Pulau Tioman presented in type of test wall (agglutinated, calcareous hyaline and calcareous porcelaneous), diversity indices (number of species (S), the number specimen collected (N), Pielou’s eveness (J’), Fisher’s alpha (α) and Shannon–wiener (H’) indices), functional groups and calculated FORAM index value.

Station	Type of test wall				Diversity indices				Functional group			FORAM index
	Water depth (m)	Agglutinated (%)	Calcareous hyaline (%)	Calcareous porcelaneous (%)	No. of species (S)	Shannon–Wiener (H’)	Pielou’s Evenness (J’)	Fisher’s alpha (α)	Symbiont-bearing (%)	Stress tolerant (%)	Others heterothrophic taxa(%)	
A1	7	1	90	9	17	2.36	0.63	6.43	69	10	21	7.5
A2	12	5	84	11	23	2.72	0.66	11.21	62	8	30	6.9
A3	13	1	93	7	19	2.27	0.51	7.23	77	9	14	8.1
B1	5	0	95	5	11	1.74	0.52	3.26	64	1	5	9.3
B2	10	4	94	2	14	2.03	0.54	4.60	94	1	9	9.2
B3	15	0	91	9	12	2.03	0.63	3.67	86	1	11	9.0
C1	5	0	93	7	18	2.36	0.59	6.58	59	13	26	6.7
C2	14	0	92	7	15	1.91	0.45	5.06	55	18	30	6.1
C3	14	3	84	12	15	2.26	0.64	5.20	50	20	34	5.6
D1	6	8	83	10	16	2.08	0.50	6.06	43	4	46	5.7
D2	11	2	89	9	17	2.33	0.61	6.17	62	13	21	7.1
D3	15	3	90	7	16	2.14	0.53	5.43	83	3	17	8.4
E1	5	1	89	10	20	2.34	0.52	7.82	84	4	16	8.4
E2	11	0	90	10	20	2.62	0.68	7.92	71	5	21	7.8
E3	18	2	71	27	24	2.95	0.79	11.13	14	22	58	3.0
F1	5	1	74	25	17	2.10	0.48	6.24	17	51	37	2.8
F2	16	2	63	35	20	2.54	0.63	8.19	29	24	47	4.1
F3	18	2	90	8	12	2.00	0.62	3.65	57	21	18	6.6
G1	5	8	69	23	23	2.69	0.64	9.91	24	27	52	3.6
G2	11	8	72	20	19	2.21	0.48	7.23	47	20	30	5.7
G3	18	0	89	11	14	1.90	0.48	4.51	84	5	13	8.5
H1	5	3	82	15	23	2.56	0.56	10.71	57	10	32	6.5
H2	7	1	93	6	17	1.83	0.37	6.03	91	1	14	8.8
H3	7	1	95	4	15	1.83	0.41	5.06	73	3	11	8.7
I1	6	2	85	13	22	2.63	0.63	9.28	71	7	21	7.6
I2	8	0	84	16	21	2.60	0.64	8.79	65	11	32	6.7
I3	10	1	94	5	19	2.52	0.65	7.14	73	13	13	7.8
J1	5	0	89	10	18	2.26	0.53	7.03	69	10	26	7.2
J2	9	0	97	3	15	1.83	0.42	5.06	85	6	14	8.4
J3	13	0	89	11	25	2.64	0.56	10.98	71	11	21	7.4

**Figure 1:**
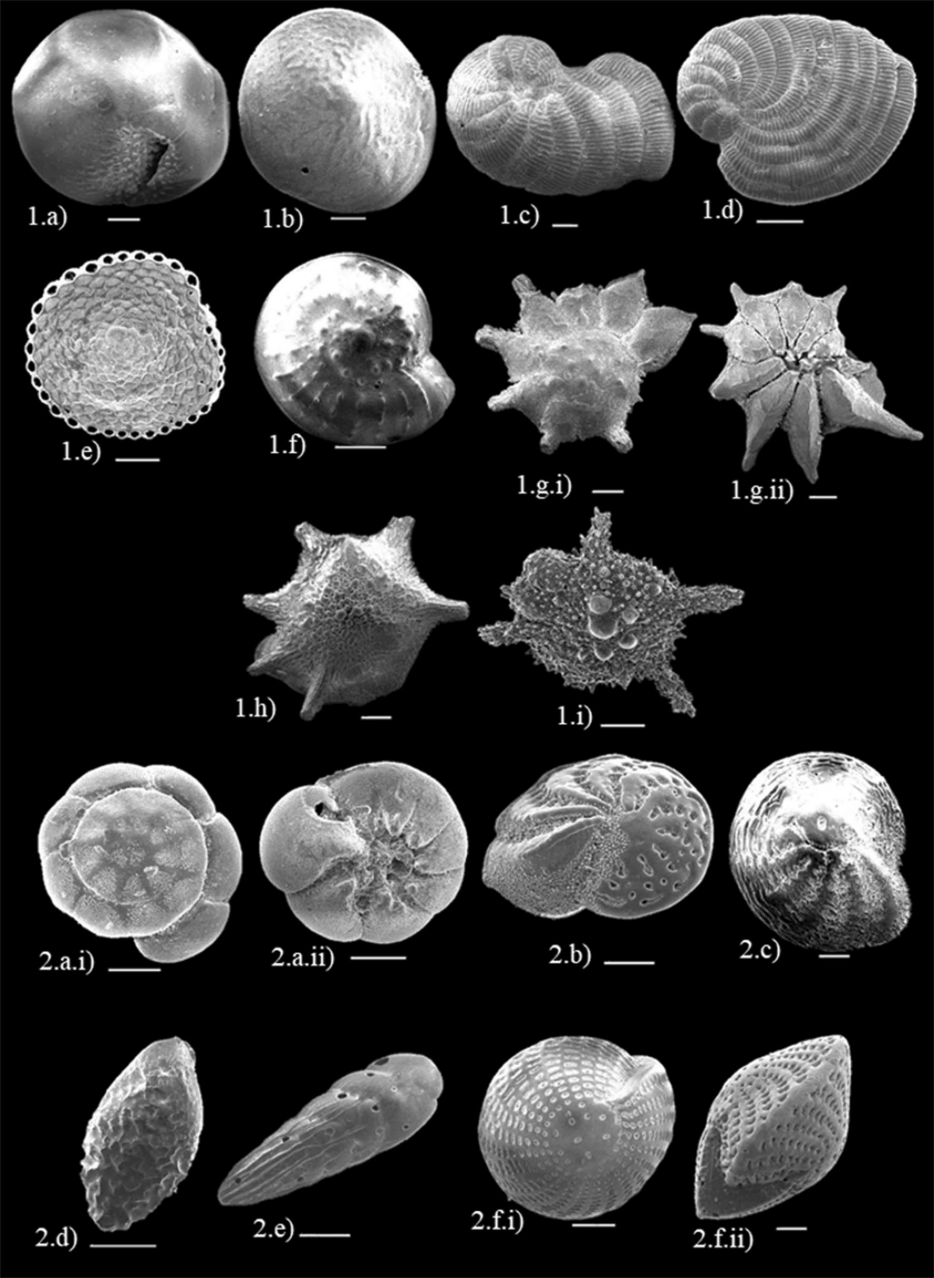
Scanning Electron Micrographs (SEM) of symbiont-bearing genus that possess relatively similar ecological needs as coral reefs. **1.a**) *Amphistegina lessonii* (100 µm × 110); **1.b**) *Amphistegina papillosa* (100 µm × 130); **1.c**) *Peneroplis pertusus* (100 µm × 100); **1.d**) *Peneroplis planatus* (200 µm × 90); **1.e**) *Sorites orbiculus* (200 µm × 80); **1.f**) *Asillina ammonoides* (500 µm × 43); **1.g.i**) Lateral side: *Pararotalia domatayi* (100 µm × 110); **1.g.ii**) Umbilical side: *Pararotalia domatayi* (100 µm × 100); **1.h**) *Calcarina gaudichaudii* (200 µm × 55); **1.i**) *Calcarina hispida* (200 µm × 80) and Scanning Electron Micrographs (SEM) of opportunist taxa (stress-tolerant)which are more resilient towards ecological changes **2.a.i**) Lateral side: *Ammonia tepida* (100 µm × 200); **2.a.ii**) Umbilical side: (100 µm × 220); **2.b**) *Elphidium crispum* (100 µm × 160); **2.c**) *Parrelina hispidula* (100 µm × 120); **2.d**) *Bolivina vadescens* (100 µm × 250); **2.e**) *Bolivina sabahensis* (100 µm × 220); **2.f.i**) Lateral side: *Cellanthus craticulatus* (200 µm × 90); **2.f.ii**) Edge side: *Cellanthus craticulatus* (100 µm × 120).

Based on the functional groups, the symbiont-bearing taxa, accounting for 63% of the occurrences, were the most common foraminifera found in almost all stations (Table 1). The symbiont-bearing taxa identified included *Amphistegina, Assilina, Calcarina, Coscinospira, Dendritina, Euthymonaca, Heterostegina, Nummulites, Operculina, Pararotalia, Parasorites, Peneroplis, Sorites*, and *Spirolina* (Fig. 1). The abundant stress-tolerant genera, accounting for 12%, included *Ammonia, Bolivina, Cellanthus, Elphidium, Pararotalia*, and *Rotalia* (Fig. 1). The remaining 25% of the occurrences were contributed by other smaller heterotrophic taxa. An extremely high dominance of the symbiont-bearing group was recorded at station B2 (Kampung Mukut) at a water depth of 10.4 m. The stress-tolerant taxa, in contrast, were dominant at station F1, which is situated close to the Marine Park Centre and jetty.

The FI values from this study varied between 2.8 and 9.2, with most sites around Pulau Tioman representing a conducive environment for reef growth and recovery (FI > 5) (Table 2). However, three stations namely F1, E3, and G1 that have FI values of 2.8, 3.0, and 3.6, respectively, indicated a marginal environment for reef growth and unsuitable conditions for reef recovery.

**Table 2:** The values and interpretation of FORAM index (FI)^11^.

FI value	Interpretation
> 4	Environment conducive to reef growth
2–4	Environment marginal for reef growth and unsuitable for recovery
** < **2	Stressed condition and unsuitable for reef growth

### Sediment characteristics and environmental condition of Pulau Tioman

Most of the sediments in the study area can be classified as sandy with coarse- to medium-grained sand dominating the grain size percentages (Table 3). The study area near Kampung Mukut (B1–B3), where a fishing village is located, has coarser sediment. The study site in Batu Mambang (J1–J3) is dominated by finer sediment. The average percentage of organic matter was 3.35 ± 0.08% with a range of 1.31–5.89% (Table 3). The highest organic matter content was recorded at E3 in the vicinity of Tekek Bay, a famous tourist site for snorkeling and diving. The lowest organic matter content was documented in Gelaber (C1), a sheltered headland that receives fewer tourists.

**Table 3:** The composition of sediment grain size and organic matter around Pulau Tioman.

Stations	Grain size (%)					Organic matter (%)
	Coarse sand (500 µm)	Medium sand (250 µm)	Fine sand (125 µm)	Very fine sand (63 µm)	Mud (< 63 µm)	
A1	6.36	56.57	34.28	2.35	0.43	2.96
A2	18.97	46.00	31.35	3.34	0.34	3.21
A3	12.57	46.64	37.12	3.29	0.39	3.06
B1	98.81	0.93	0.13	0.09	0.04	3.45
B2	69.48	23.53	4.95	1.62	0.43	3.74
B3	98.95	0.71	0.19	0.12	0.03	3.22
C1	44.89	42.47	10.73	1.61	0.30	1.31
C2	73.51	16.30	9.72	0.15	0.32	1.82
C3	28.12	47.69	21.60	2.08	0.51	2.14
D1	93.21	5.55	0.94	0.21	0.09	3.26
D2	57.75	23.45	16.50	1.72	0.59	3.52
D3	85.26	12.33	2.11	0.20	0.10	3.45
E1	82.66	13.70	2.76	0.55	0.33	3.35
E2	39.10	20.34	24.54	11.91	4.12	3.75
E3	77.80	10.00	6.30	5.43	0.47	5.89
F1	87.05	3.61	3.37	5.84	0.13	2.19
F2	7.05	10.35	44.63	28.70	9.27	3.58
F3	32.14	21.48	36.02	5.91	4.46	4.13
G1	90.02	9.30	0.58	0.09	0.01	3.58
G2	87.30	9.22	1.29	0.18	2.01	3.51
G3	33.76	34.48	29.31	2.19	0.26	3.28
H1	51.91	20.20	20.17	6.74	0.97	3.70
H2	68.99	16.47	10.15	3.42	0.96	3.50
H3	90.04	7.60	1.70	0.42	0.24	3.75
I1	36.67	9.09	50.23	0.02	3.99	3.80
I2	23.76	4.85	62.46	0.18	8.75	3.29
I3	1.52	17.79	68.07	12.56	0.07	2.97
J1	3.50	42.23	49.68	4.55	0.03	3.75
J2	49.52	46.91	3.26	0.27	0.04	3.48
J3	5.63	28.25	47.97	18.09	0.06	3.91

The average bottom water temperature around Pulau Tioman was 29 °C with salinity of 33 PSU. The concentration of dissolved oxygen (DO) varied between 4.5 and 6.9 mg/L with the highest amount of DO recorded at B1 (Kampung Mukut). Meanwhile the water pH recorded was between 8.1 and 8.7.

### Statistical analysis of benthic foraminiferal assemblages

**Figure 2:**
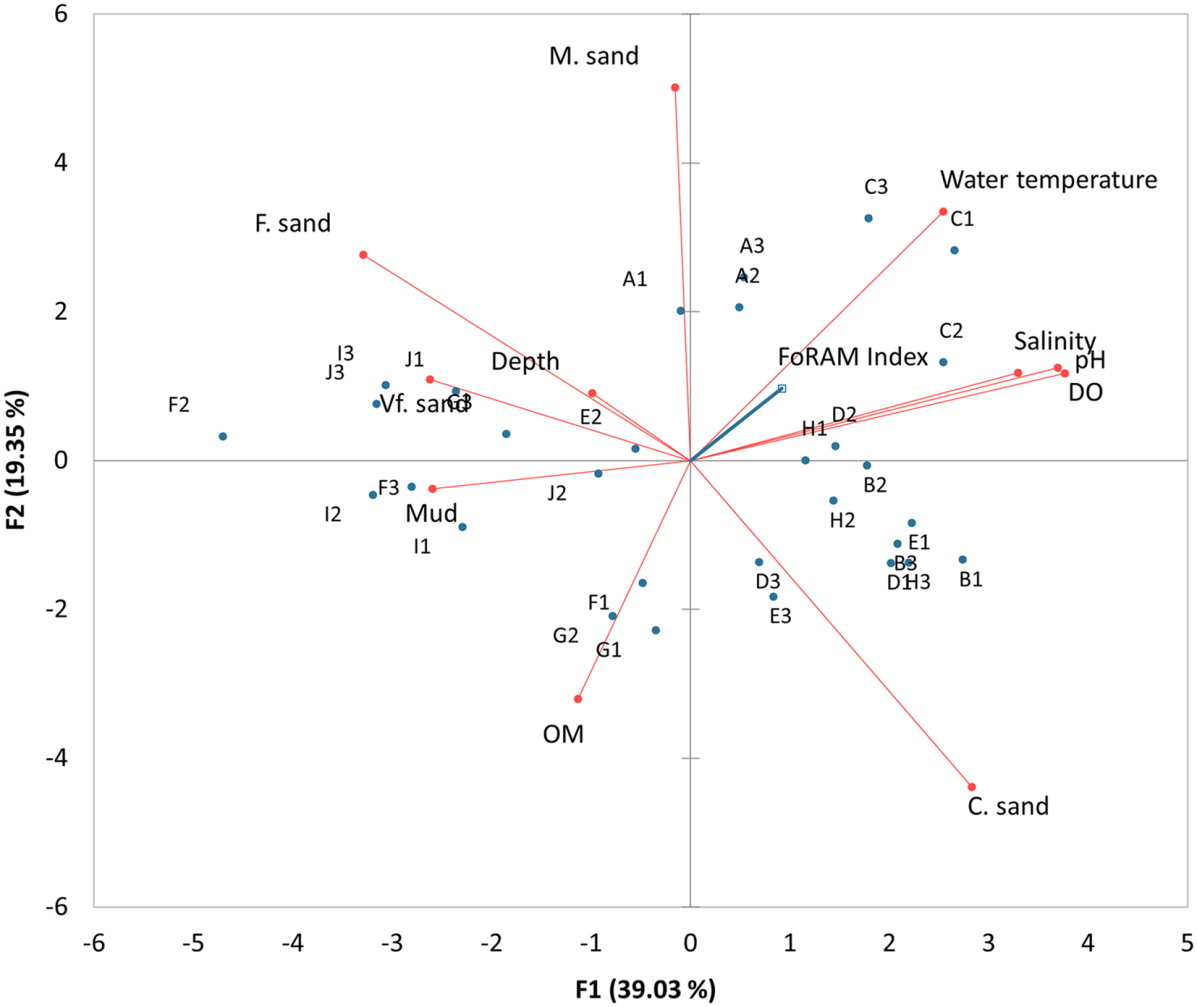
Principal component analysis (PCA) of sediment and water quality and FI data from study area around Pulau Tioman. The variation explained by both axes 1 and 2 is 58.38%. OM-organic matter; C. sand-coarse sand; DO- Dissolved oxygen; M. sand- Medium sand; F. sand- Fine sand; Vf, sand- Very fine sand.

The first and second axes of the Principal Component Analysis (PCA) explained 58.38% of the variations between the water quality and sediment quality variables (Fig. 2). In addition, the supplementary variables for the FI values indicated a positive correlation between these values and the dissolved oxygen, pH, salinity, and water temperature. Meanwhile, the FI values showed a negative correlation with organic matter, fine sand particles, and the mud composition.

**Figure 3:**
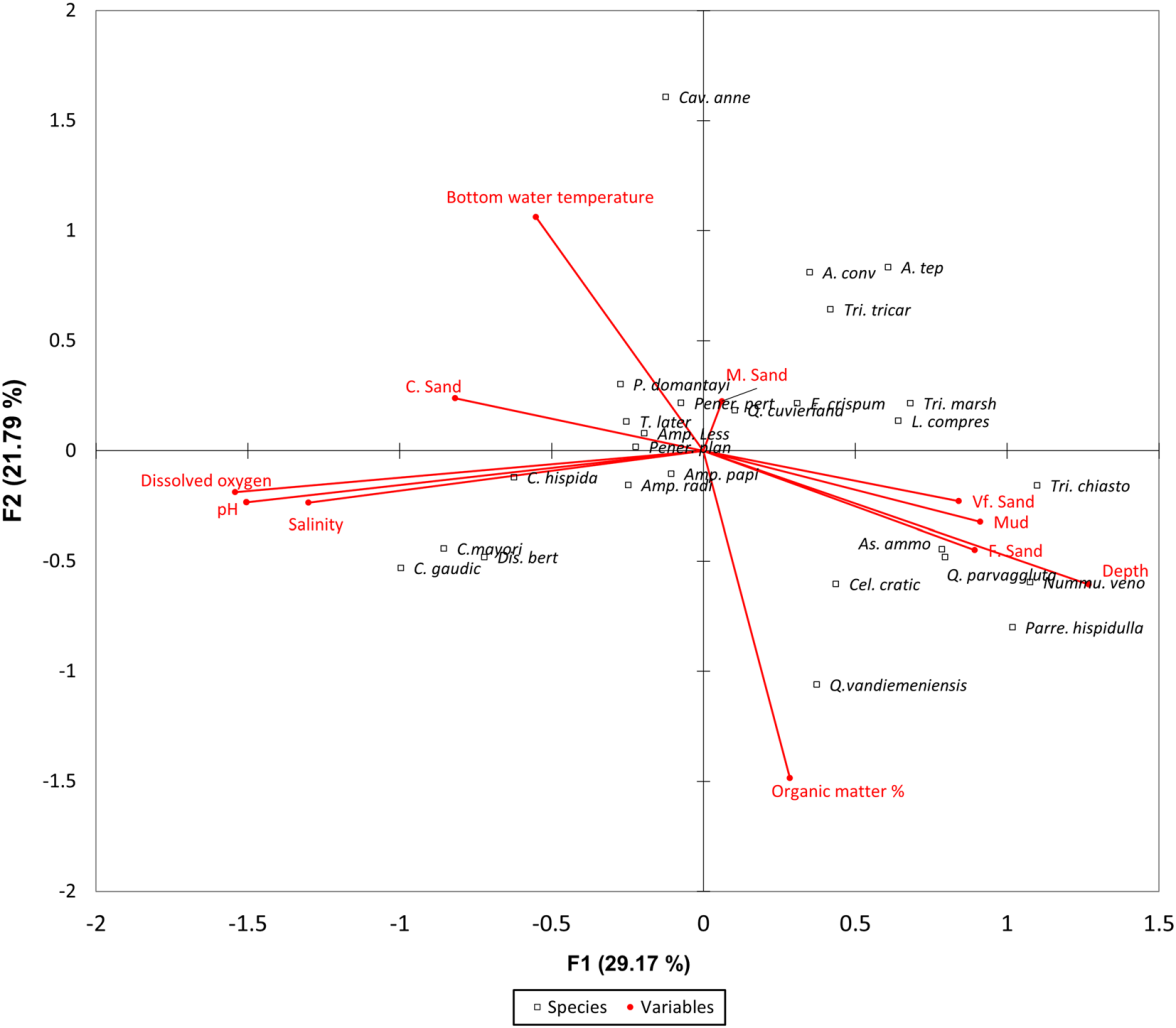
Species-environmental biplot based on Canonical Correspondence Analysis (CCA) of data collected from Pulau Tioman. The total variance of both axes 1 and 2 is (50.96%). (OM-organic matter; C. sand-coarse sand; DO- Dissolved oxygen; M. sand- Medium sand; F. sand- Fine sand; Vf, sand- Very fine sand) (*A. tep* = *Ammonia tepida*; *Amp. less* = *Amphistegina lessoni*; *Amp. papi* = *Amphistegina papillosa*; *Amp. radi* = *Amphistegina radiata*; *As. ammo* = *Assilina ammonoides*; *C. gaudic* = *Calcarina gaudichaudii*; *C. hispida* = *Calcarina hispida*; *C. mayori* = *Calcarina mayori*; *Cav. Anne * = *Cavarotalia annectens*; *Cel. cratic* = *Cellanthus craticulatus*; *Dis. bert* = *Discorbinella bertheloti*; *E. crispum* = *Elphidium crispum*; *L. compres* = *Lachlanella compressiostoma*; *Nummu. veno* = *Nummulites venosus*; *P. domantayi* = *Pararotalia domantayi*; *Pener. hispidulla* = *Parrelina hispidulla*; *Pener. pert* = *Peneroplis pertusus*; *Pener. plan* = *Peneroplis planatus*; *Q. vandiemeniensis* = *Quinqueloculina vandiemeniensis; Q. cuvieriana* = *Quinqueloculina cuvieriana*; *Q. parvaggluta* = *Quinqueloculina parvaggluta*; *T. later* = *Textularia lateralis*; *Tri. marsh* = *Triloculina marshallana*; *Tri. tricar* = *Triloculina tricarinata*; *Tri. chiasto* = *Triloculinella chiastocytis*).

**Figure 4:**
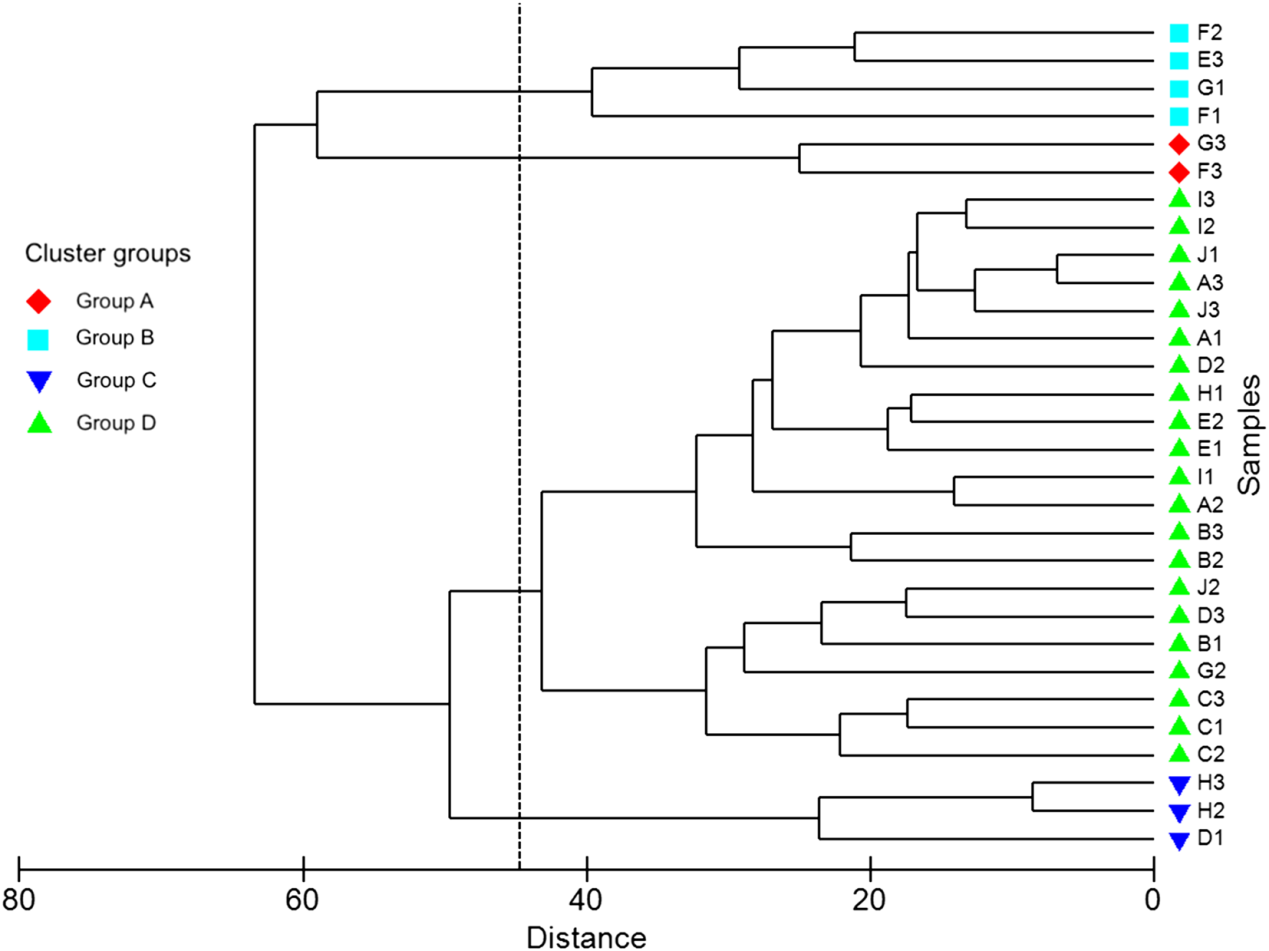
Dendogram produced by cluster analysis based on the complete linkage of benthic foraminiferal assemblages around Pulau Tioman. The stations in Pulau Tioman are divided into four groups (i.e., Group **A**, **B**, **C** and **D**) represented by the symbols are based on the distribution of benthic foraminifera assemblages. The distance represents Euclidean distance.

The Monte Carlo permutation test showed that the foraminifera species are linearly related to the water quality and sediment quality data (*p* < 0.05). The axis-1 of Canonical Correspondence Analysis (CCA) explained 29.1% of the variance between the species and environmental variables (eigenvalue: 0.29) and axis-2 explained 21.8% (eigenvalue: 0.22; Fig. 3). Cluster analysis was conducted to determine the similarities between all the sampling sites based on the number of foraminifera species found around Pulau Tioman. The Q-mode analysis revealed four major groups of benthic foraminiferal assemblages: Group A, Group B, Group C, and Group D (Fig. 4). Group A consisted of two stations (Stations F3 and G3). The foraminiferal assemblages in this group were characterized by a high average abundance of *Nummulites venosus* (31%) and *Assilina ammonoides* (22%). Group B consisted of Stations E3, F2, G1, and F1. The species that dominated the assemblages in this group was *Ammonia tepida* with an average relative abundance of 17%*.* Group C consisted of Stations D1, H2, and H3, and was dominated by *Calcarina gaudichaudii* (34%) and *Amphistegina lessoni* (23%). Group D consisted of the remaining 21 stations (Stations A1, A2, A3, B1, B2, B3, C1, C2, C3, D2, D3, E1, E2, G2, H1, I1, I2, I3, J1, J2, and J3). The common species identified for the Group D area were *Amphistegina lessoni* (27%) and *Amphistegina papillosa* (8%).

## Discussion

### Foraminiferal assemblages in Pulau Tioman

The foraminifera assemblages in Pulau Tioman are dominated by rotaliid genera, such as *Amphistegina*, *Calcarina*, *Operculina*, and *Peneroplis*, with most species having a symbiotic relationship with diatom or algae (Appendix 1), which is similar to worldwide reef foraminifera distributions^16–18^. The foraminifera diversity around Pulau Tioman is slightly higher (H′ = 1.8–3.0) than those reported from the fringing reef environment in Brazil^19^. *Amphistegina lessoni* and *Calcarina gaudichaudii*, which are among the most widespread species found in Indo-Pacific waters^17^, are highly abundant and common in Pulau Tioman. *Amphistegina lessoni*, for instance, occurs at all stations around Pulau Tioman, except in F3 (Mesoh), where conditions of high water depths (> 18 m), algal distribution, and turbidity exist, which may restrict their presence^20^. Despite relatively lower abundances (4–55%) compared to that reported in the study on the northern atoll in the South China Sea^21^, the calcareous porcelaneous group (*Triloculina, Quinqueloculina*, and *Lachlanella*) contributed to > 50% of the total foraminiferal assemblage in Pulau Tioman. In Mesoh, where the water depth was > 15 m and slightly murky, there was a significant increase in porcelaneous representatives, such as *Lachlanella compressiostoma* (35%), *Triloculina tricarinata* (23%), and *Triloculinella chiastocytis* (21%). This finding supports the observations reported in Uthicke et al.^8^ in the Great Barrier Reef, where a high abundance of miliolids was recorded in turbid waters. *Triloculina* was common in the Dongsha Atoll in the northern South China Sea, but their abundance was recorded to be < 20%^21^.

*Amphistegina* and *Calcarina* are common in reef environments worldwide^22,23^, especially in Southeast Asia^9,24,25^. Most living *Amphistegina* can be found attached to reef substrates, with less of a presence in sediment, and usually occur in high abundance in coral reef environments^9^. Owing to their ecological requirements and distribution, foraminifera assemblages are among the best candidates to monitor reef health. Opportunistic taxa, especially *Ammonia*, *Elphidium*, and *Bolivina*, are among those that are tolerant to environmental variations^11,13^. Hence, typically polluted marine conditions, with organic matter enrichment and reduced dissolved oxygen, allow these groups to opportunistically increase in abundance^15^.

In addition, the CCA indicated that environmental variables, such as the concentration of dissolved oxygen (mg/L), pH, salinity, and organic matter composition, had the most significant effect on the distribution of benthic foraminifera around Pulau Tioman (Fig. 3). Symbiont-bearing species, such as *Calcarina* spp., correlated well with the increase in the dissolved oxygen concentration, salinity, and pH. Previously, Prazeres et al.^26^ suggested the need for calibration of the FI for the Indo-west Pacific where Calcarinidae occur. This is because *Calcarina* spp. usually thrive even in mesothrophic reefs^20^. Despite this concern, our study indicated that not only *Calcarina* spp. exhibits a good correlation with the water quality. Their relative abundance was significantly higher (> 15%) in Salang Bay, an area adjacent to where Shahbudin et al.^3^ reported the highest percentages of live coral coverage. Therefore, based on the species-environment relationship assessment, we speculate that calibration of the FI was not necessary for our study area.

### Ecological interpretation of FORAM index

The increase in construction and land reclamation activities around the east coast region has exposed many coral reef areas to high rates of sedimentation and a consequent reduction of the diversity of live corals including those in Pulau Tioman^3^. Additionally, active tourism related activities, such as trampling by divers or snorkelers and resuspension of sediment by boats has increased the mortality rate of corals^27,28^.

A majority of the FI values obtained in the waters around Pulau Tioman were greater than 4, which indicate that the water quality is favorable for reef growth and recovery^8,14^. Despite the excellent performance of the FI in numerous studies^8,16,29,30^, Prazeres et al.^26^ have raised some concerns on the application of this index in new study areas. Therefore, to address this concern and reduce the bias associated with the application of the FI index, we performed PCA. Based on the PCA results (Fig. 2), the FI values in this study showed a positive correlation with good water qualities and a negative correlation with organic matter enrichment. Additionally, the FI values observed in east Pulau Tioman were > 5, which were higher than those observed in the west. This study, therefore, indicates that the reefs in east Pulau Tioman are more likely to survive and recover from future bleaching events compared to those on the west. Our finding are comparable to the study by Shahbudin et al.^3^, which reported that coral coverage in east Pulau Tioman was better than that in the west, where a higher percentage of dead corals was observed. The degraded reef conditions along the west coast of Pulau Tioman has been associated with rapid coastal development^31^, active tourism^32^, and boating activities^3^.

Three stations (i.e., E3, F1, and G1) that recorded lower FI values (FI < 4) in this study are famous diving and snorkeling sites, and their reef conditions are similar to those established by Shahbudin et al.^3^ and Akmal et al.^33^. These three stations are located near the jetty of the Pulau Tioman Marine Park, which also serves as the route for boat journeys to tourist spots for snorkeling and SCUBA diving activities. These relatively higher anthropogenic activities have led to an increase in the organic matter and nutrient concentrations in the water, which have created a less favorable environment for symbiont-bearing foraminifera and allowed stress-tolerant taxa to dominate^8,11^.

To understand the similarity of the foraminifera assemblages around Pulau Tioman, a Q-mode cluster analysis was conducted. The results indicated that the benthic foraminifera assemblages can be classified into four major groups (i.e., Group A, Group B, Group C, and Group D) (Fig. 4). Three of these groups (Group A, Group B, and Group C) represent foraminifera assemblages found on the western side of the island. Group A represents deep water conditions dominated by *Numulites venosus* and *Operculina ammonoides*. Both symbiont-bearing species that belong to the Nummulitidae family have been reported to have better growth rates in regions with low light^34^, thereby explaining their increase in abundance at the deeper reef slope area (> 18 m water depth) in west Pulau Tioman. The calculated FI for Group A (FI > 6) shows that the water quality conditions within these stations (F3 and G3) can be classified as oligotrophic, which is optimal for reef recovery^11,26^. Hence, despite the intensity of diving activity in Mesoh and Panuba Bay, the increased distance from the shoreline has reduced the anthropogenic impacts on coral reefs and supported healthy coral growth and recovery^35^. Meanwhile, Group B recorded a significant increase in the porcelaneous foraminifera group, with a higher abundance of stress-tolerant taxa, such as *Ammonia tepida.* In west Pulau Tioman, the high average composition of organic matter (3.81%) in the sediment serves as a food source for heterotrophic taxa and increases the number of stress tolerant species, such as *Ammonia*. The FI values for this group varied between 2.7 and 4.0, with the lowest values recorded in the vicinity of Mesoh, where high snorkeling and diving activities occur. The sheltered beach in Mesoh is among the most popular sites for locals and tourists to enjoy swimming and snorkeling. Based on the observations during field sampling, the water in F1 (Mesoh) is more turbid with numerous coral fragments present close to the shoreline. Group C represents foraminifera assemblages that are distributed in shallow waters (6–7 m depth) in west Pulau Tioman. This group has a relatively lower quantity of stress-tolerant taxa (< 5%), but a relatively higher quantity of heterotrophic species, such as *Eponides*, *Discorbinella*, and *Textularia*. The presence of abundant sources of food may have promoted the increase in the number of heterotrophic taxa but they are limited by the dominance of stress-tolerant taxa^11,13^. Finally, group D represents most of the stations located in east Pulau Tioman with an FI of 6–9, indicating good water conditions for reef growth and recovery. The reduced coastal development activities observed in east Pulau Tioman may have decreased the impact of sedimentation and allowed a greater diversity of live corals to thrive^3^.

This study indicates that the FI is an excellent low-cost monitoring tool that can aid in assessments of water quality surrounding coral reefs. This finding also implies that the FI can be used together with the coral reef health index to determine the conditions and status of coral reefs in the region. Similarly, these indices can be applied to other coral reef ecosystems around Malaysia to determine their health. The range of FI values reported here shows that most of the sampling sites around Pulau Tioman are conducive to coral reef growth and recovery after exposure to any future bleaching events or temporary damages to the ecosystem. Several sites (e.g., Mesoh and Tekek bays) with reduced FI values may not provide optimum conditions for reef recovery. Hence, marine park managers must closely monitor these touristic sites to prevent further coral reef deterioration. The carrying capacity of this ecosystem with respect to visitors and tourists at the Tioman Marine Park should be reviewed to determine optimum conditions for coral reef health, as indicated by the FI results.

## Methods

### Study site

**Figure 5:**
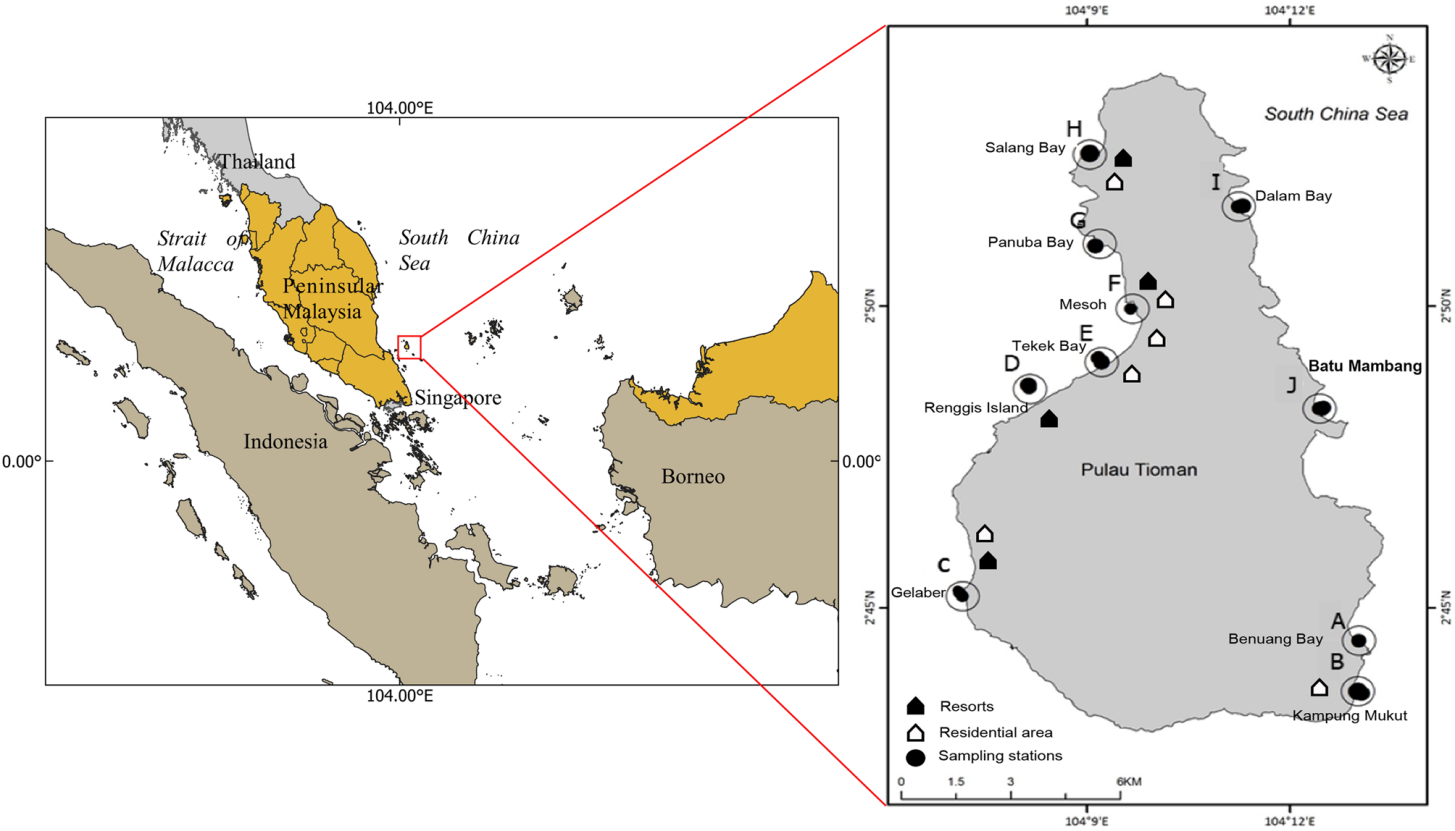
The study area shows (**A**) the map of Peninsular Malaysia with a box marking Tioman Marine Park. (**B**) The distribution of sampling stations, resorts, and residential areas on the east and west coast of Pulau Tioman, Malaysia. (Map produce using the Free and Open Source QGIS 3.16.).

The study was conducted at Pulau Tioman, Pahang, Malaysia (Fig. 5). Pulau Tioman is a tropical island situated in the southern South China Sea (SSCS) and is surrounded by extensive coral coverage, which hosts various marine species^36^. The island is influenced by two monsoonal systems: the northeast (November–March) and southwest monsoons (April–August)^37^. The maximum wave height during the northeast monsoon is ~ 4 m while during the southwest monsoon the height is < 1 m^38,39^. Pulau Tioman is surrounded by numerous coral reefs, with approximately 57–59 genera of hard corals distributed around the island^3^. *Acropora*, *Montipora*, and *Porites* are among the most common coral genera present around Pulau Tioman^3,27^. The extensive reef ecosystem surrounding Pulau Tioman is among the reasons why this island has been established as a National Marine Park by the Malaysian government^40^. The beauty of the island has attracted tourism-related activities since 1990^41^. The sheltered west coast of Pulau Tioman (Fig. 5) has become a suitable site for snorkeling and diving activities, as compared with the east side of the island. Therefore, the west coast of Pulau Tioman receives more tourists and is substantially more developed, with numerous resorts and housing areas distributed along the coast^3^. Tourist and recreational diving activities and coastal development may pose a threat to the surrounding reef ecosystem by reducing its resilience to climate change^6^. Therefore, the most viable management approach in the face of climate change is to reduce and monitor local stressors, such as coastal pollution^4,42^.

### Sample collection

To assess the marine environmental health of Pulau Tioman, samples were collected from the coastal waters of the west and east sides of the island. A total of 10 sampling sites were selected based on the intensity of human activities and development along the coast of Pulau Tioman (Fig. 5). A transect of three sampling points was setup at each sampling site for a total of 30 sampling points around Pulau Tioman (Table 4). At each sampling site, three samples were collected along the 100-m transect laid perpendicular to the shore toward the reef slope. Along each transect, one sample was collected at 50-m intervals. Bulk sediment samples were collected by SCUBA divers using a scoop, which were stored in labelled plastic bags. Parameters, such as the water salinity (PSU), temperature (°C), pH, and water depth (m), were obtained in situ at each sampling station using a Hydrolab Quanta Multiparameter operated from a small vessel. All of the collected samples were transported to the Pulau Tioman marine park station in Mesoh for sorting. Sediment samples were divided into two components for foraminiferal and sedimentological analyses. Approximately 30 cm^3^ of sediments were subsampled from the bulk samples for foraminiferal analysis and were fixed with 4% buffered formalin^43^. The remaining sediment samples were stored in zip-lock plastic bags, labelled, and brought back to the Central Laboratory of Universiti Malaysia Terengganu for sediment grain size analysis.

**Table 4:** Brief description and coordinate of all 30 sampling stations around the coastal waters of Pulau Tioman. All location names are written in bold in the description column.

Description of locations	Station	Coordinate	
		Longitude	Latitude
**Benuang** Low number of snorkelling and SCUBA diving activity	A1	2° 44′27.4′′ N	104° 13′01.3′′ E
	A2	2° 42′27.7′′ N	104° 13′04.9′′ E
	A3	2° 42′28.9′′ N	104° 13′06.3′′ E
**Kampung Mukut** Fisherman village, not a snorkelling and SCUBA diving site	B1	2° 43′37.4′′ N	104° 13′00.3′′ E
	B2	2° 43′36.2′′ N	104° 13′01.9′′ E
	B3	2° 43′35.2′′ N	104° 13′03.5′′ E
**Gelaber** Not a snorkelling and SCUBA diving site	C1	2° 45′11.6′′ N	104° 07′09.7′′ E
	C2	2° 45′15.5′′ N	104° 07′07.1′′ E
	C3	2° 45′17.7′′ N	104° 07′05.2′′ E
**Renggis Island** High number of snorkelling and SCUBA diving activities at this site	D1	2° 48′37.4′′ N	104° 08′09.6′′ E
	D2	2° 48′39.0′′ N	104° 08′08.8′′ E
	D3	2° 48′41.6′′ N	104° 08′08.3′′ E
**Tekek Bay** Snorkelling and SCUBA diving site, boating route, Jetty	E1	2° 49′04.4′′ N	104° 09′13.2′′ E
	E2	2° 49′08.2′′ N	104° 09′10.4′′ E
	E3	2° 49′11.5′′ N	104° 09′08.2′′ E
**Mesoh** Marine Park Centre, Jetty, snorkelling and SCUBA diving site	F1	2° 49′57.9′′ N	104° 09′42.4′′ E
	F2	2° 49′57.4′′ N	104° 09′40.7′′ E
	F3	2° 49′57.1′′ N	104° 09′38.7′′ E
**Panuba Bay** Snorkelling and SCUBA diving site, boating route	G1	2° 51′01.9′′ N	104° 09′11.4′′ E
	G2	2° 51′00.8′′ N	104° 09′09.4′′ E
	G3	2° 50′59.6′′ N	104° 09′07.5′′ E
**Salang Bay** Not a snorkelling and SCUBA diving site	H1	2° 52′30.4′′ N	104° 09′02.6′′ E
	H2	2° 52′31.9′′ N	104° 09′02.5′′ E
	H3	2° 52′33.5′′ N	104° 09′02.7′′ E
**Dalam Bay** Not a snorkelling and SCUBA diving site	11	2° 51′38.8′′ N	104° 11′14.5′′ E
	I2	2° 51′38.9′′ N	104° 11′16.8′′ E
	I3	2° 51′39.5′′ N	104° 11′18.2′′ E
**Batu Mambang** Not a snorkelling and SCUBA diving site	J1	2° 48′18.1′′ N	104° 12′26.2′′ E
	J2	2° 48′18.1′′ N	104° 12′28.3′′ E
	J3	2° 48′19.1′′ N	104° 12′30.1′′ E

### Laboratory analysis

The sediment samples for foraminiferal analysis were gently washed under running tap water over a 63-µm mesh sieve^11^. The residue on the 63-µm sieve was carefully transferred into a pre-labelled weighing boat before being dried in the oven at 40–50 °C overnight. The dried samples were later stored in plastic bags for analysis. Foraminiferal specimens were handpicked using a fine brush under a stereomicroscope. A total of 300 optimally preserved foraminifera tests were picked from every station^24^. If the samples contained less than 300 foraminifera, all of the specimens were picked^24^. Then, the specimens were sorted and mounted on micropaleontological cardboard slides. Foraminifera identification was performed based on the methods reported in Loeblich & Tappan^44^ and other regional taxonomic manuscripts^24,45,46^.

The grain size analysis was conducted based on the dry-sieving method by Folk^47^. Approximately 100 g of dried sediment samples were sieved using a sieve set containing 4000-, 2000-, 1000-, 500-, 250-, 125-, and 63-µm sieves and the percentage of each sediment size fraction was determined. The organic matter content in the sediments was determined using the loss on ignition (LOI) method^48^. Prior to heating, 5 g of each sediment sample was weighed. The samples were gradually heated until they reached 500–550 °C, followed by ignition for 4 h, cooled to room temperature (30 °C), and the final weight was recorded. The percentages of organic matter were calculated based on the formula provided in Heiri et al.^49^.

### Data and statistical analyses

To avoid using reworked samples, only foraminiferal species with a relative abundance of more than 2% were maintained for further statistical analysis. Indices, such as Fisher’s alpha diversity index (α), Shannon–Wiener species diversity (H′), and species evenness of Pielou (J′), were analyzed using the PAST (PAleontological STatistics) software version 3. Cluster analysis was performed to simplify the large datasets for easy recognition^50^. The Principal Component Analysis (PCA) was performed to investigate the relationships between the FI values and the sediment and water quality around Pulau Tioman. In addition, to investigate the relationships between the foraminifera species and the environment, canonical correspondence analysis (CCA) with a Monte Carlo permutation test was performed^51^. Foraminifera species with a relative abundance of > 10% in at least one sample were selected for the CCA and permutation test.

### FORAM index

The FI was used to determine the health status of the reef at Pulau Tioman. The foraminifera species collected around Pulau Tioman were identified and segregated into functional groups (Table 5), as suggested by Hallock et al.^11^ and Carnahan et al.^29^. The calculation of the FI was based on the equation proposed by Hallock et al.^11^. The values obtained from the calculation were interpreted based on Table 2.

**Table 5:** Functional Groups assigned to benthic foraminifera used in coral reef assessment^11,29^.

Functional group	Order	Family	Genus	Distribution
Symbiont-bearing	Rotaliida	Amphisteginidae	*Amphistegina*	Circumtropical
		Calcarinidae	5 genera	Indo-Pacific
		Nummulitidae	*Heterostegina*	Circumtropical
			3 other genera	Indo-Pacific
	Miliolida	Miliolida	*Alveolinella*	Indo-Pacific
			*Borelis*	Circumtropical
		Peneroplidae	Several genera	Circumtropical
		Soritidae	Sorites	Circumtropical
			*Amphisorus*	Circumtropical
			3 genera	Caribbean
			*Marginopora*	Indo-Pacific
Opportunistic* (stress-tolerant)	Trochamminida	Trochamminidae	Several genera	Cosmopolitan
	Textulariida	Lituolidae	Several genera	Cosmopolitan
	Buliminida	Bolivinidae	Several genera	Cosmopolitan
		Buliminidae	Several genera	Cosmopolitan
	Rotaliida	Rotaliidae	*Ammonia*	Cosmopolitan
		Elphidiidae	*Elphidium*	Cosmopolitan
Other (Heterotrophic) Small Taxa	Miliolida	Most except larger taxa noted above		Cosmopolitan
	Rotaliida	Most except those noted above		Cosmopolitan
	Textulariida	Most		Cosmopolitan
	Other	Most		Cosmopolitan

*Full range of opportunistic genera under local conditions is not well known.

## Supplementary Information

Supplementary Information.

## Data Availability

All data generated or analyzed during this study are included in this article (and its Supplementary Information files).
