# Supplementary material for: Reef foraminifera as bioindicators of coral reef health in southern South China Sea
Source: Sci Rep. 2021 Apr 26;11:8890. doi: 10.1038/s41598-021-88404-3 (PMC8076234; doi:10.1038/s41598-021-88404-3)
Supplement: Supplementary file 1 — Supplementary Information. [file 41598_2021_88404_MOESM1_ESM.docx]

**Reef foraminifera as bioindicators of coral reef health in southern South China Sea**

**Authors**

Aishah Norashikin Abdul A’ziz^1^

Email: [aishahnorashikin96@gmail.com](mailto:aishahnorashikin96@gmail.com)

Fatin Izzati Minhat^1,2^

Email: [fatinminhat@umt.edu.my](mailto:fatinminhat@umt.edu.my)

Hui-Juan Pan^3^

Email: [hjpan76@gmail.com](mailto:hjpan76@gmail.com)

Hasrizal Shaari^1,2^

Email: [riz@umt.edu.my](mailto:riz@umt.edu.my)

Wan Nurzalia Wan Saelan ^1,2^

Email: [wan.nurzalia@umt.edu.my](mailto:wan.nurzalia@umt.edu.my)

Nazihah Azmi^1^

Email: [nazihahazmi28@gmail.com](mailto:nazihahazmi28@gmail.com)

Omar Abdul Rahman Abdul Manaf^1^

Email: [omarabdrahman96@gmail.com](mailto:omarabdrahman96@gmail.com)

Md Nizam Ismail^4^

Email: mdnizam@nre.gov.my

**Affiliations**

^1^Faculty of Science and Marine Environment, Universiti Malaysia Terengganu, 21030 Kuala Nerus, Terengganu, Malaysia.

^2^Institute of Oceanography and Environment, Universiti Malaysia Terengganu, 21030 Kuala Nerus, Terengganu, Malaysia.

^3^Institute of Earth Sciences, College of Ocean Science and Resource, National Taiwan Ocean University, Keelung, Taiwan

^4^Fisheries Research Institute, 11960 Batu Maung, Pulau Pinang, Malaysia.

***Corresponding author details**

Fatin Izzati Minhat

fatinminhat@umt.edu.my

Appendix 1 List of benthic foraminifera species and their relative abundance in all 30 stations around Pulau Tioman.

| **Species** | A1 | A2 | A3 | B1 | B2 | B3 | C1 | C2 | C3 | D1 | D2 | D3 | E1 | E2 | E3 | F1 | F2 | F3 | G1 | G2 | G3 | H1 | H2 | H3 | I1 | I2 | I3 | J1 | J2 | J3 |
| --- | --- | --- | --- | --- | --- | --- | --- | --- | --- | --- | --- | --- | --- | --- | --- | --- | --- | --- | --- | --- | --- | --- | --- | --- | --- | --- | --- | --- | --- | --- |
| *Ammonia convexa* | 0 | 0 | 0 | 0 | 0 | 0 | 1 | 2 | 5 | 0 | 0 | 0 | 0 | 0 | 2 | 0 | 0 | 0 | 9 | 3 | 2 | 0 | 0 | 0 | 0 | 1 | 0 | 0 | 0 | 0 |
| *Ammonia supera* | 0 | 0 | 1 | 0 | 0 | 0 | 3 | 2 | 0 | 0 | 0 | 0 | 0 | 0 | 0 | 0 | 2 | 0 | 0 | 0 | 0 | 3 | 0 | 0 | 0 | 0 | 4 | 0 | 0 | 0 |
| *Ammonia tepida* | 0 | 0 | 0 | 0 | 0 | 0 | 3 | 2 | 7 | 1 | 0 | 0 | 0 | 0 | 5 | 39 | 11 | 5 | 14 | 15 | 1 | 0 | 0 | 0 | 1 | 5 | 0 | 2 | 1 | 5 |
| *Amphistegina lessoni* | 18 | 14 | 29 | 41 | 19 | 19 | 25 | 39 | 27 | 16 | 26 | 40 | 33 | 19 | 2 | 2 | 2 | 0 | 11 | 36 | 18 | 21 | 25 | 29 | 14 | 20 | 19 | 28 | 48 | 27 |
| *Amphistegina papillosa* | 18 | 9 | 16 | 4 | 10 | 12 | 8 | 2 | 2 | 1 | 14 | 7 | 4 | 4 | 0 | 1 | 0 | 1 | 1 | 1 | 2 | 3 | 2 | 3 | 7 | 9 | 11 | 12 | 10 | 13 |
| *Amphistegina radiata* | 10 | 9 | 12 | 13 | 10 | 19 | 6 | 0 | 0 | 1 | 6 | 6 | 6 | 13 | 0 | 0 | 0 | 0 | 1 | 2 | 3 | 3 | 3 | 3 | 5 | 6 | 13 | 9 | 6 | 5 |
| *Assilina ammonoides* | 5 | 9 | 6 | 2 | 3 | 11 | 0 | 0 | 3 | 0 | 2 | 6 | 1 | 6 | 10 | 2 | 18 | 23 | 0 | 1 | 21 | 1 | 0 | 2 | 8 | 11 | 10 | 6 | 4 | 6 |
| *Bolivina vadescens* | 0 | 9 | 0 | 0 | 0 | 0 | 0 | 0 | 0 | 1 | 0 | 0 | 0 | 0 | 3 | 0 | 0 | 0 | 0 | 0 | 0 | 0 | 0 | 0 | 0 | 0 | 0 | 0 | 0 | 0 |
| *Calcarina gaudichaudii* | 0 | 9 | 0 | 2 | 16 | 1 | 0 | 0 | 0 | 26 | 4 | 0 | 11 | 10 | 0 | 0 | 0 | 0 | 1 | 2 | 0 | 15 | 41 | 36 | 1 | 0 | 0 | 0 | 1 | 4 |
| *Calcarina hispida* | 2 | 9 | 1 | 17 | 24 | 18 | 3 | 2 | 1 | 0 | 0 | 0 | 3 | 2 | 0 | 0 | 0 | 0 | 0 | 0 | 0 | 1 | 1 | 3 | 6 | 1 | 3 | 0 | 0 | 2 |
| *Calcarina mayori* | 0 | 9 | 1 | 3 | 3 | 0 | 0 | 0 | 0 | 0 | 0 | 0 | 5 | 5 | 0 | 0 | 0 | 0 | 0 | 0 | 0 | 1 | 6 | 5 | 0 | 0 | 0 | 0 | 2 | 0 |
| *Cavarotalia annectens* | 3 | 9 | 0 | 0 | 0 | 0 | 18 | 19 | 9 | 0 | 0 | 0 | 1 | 0 | 0 | 0 | 0 | 0 | 12 | 0 | 0 | 0 | 0 | 0 | 0 | 0 | 0 | 0 | 6 | 1 |
| *Cellanthus craticulatus* | 0 | 9 | 0 | 0 | 0 | 1 | 0 | 0 | 3 | 1 | 11 | 2 | 3 | 5 | 4 | 0 | 2 | 12 | 1 | 2 | 2 | 0 | 1 | 2 | 0 | 0 | 2 | 0 | 2 | 1 |
| *Discorbinella bertheloti* | 0 | 9 | 0 | 0 | 0 | 0 | 0 | 0 | 0 | 11 | 0 | 0 | 0 | 0 | 4 | 0 | 0 | 0 | 0 | 0 | 0 | 0 | 0 | 0 | 0 | 1 | 1 | 0 | 0 | 0 |
| *Dendritina ambigua* | 0 | 9 | 0 | 0 | 0 | 0 | 0 | 0 | 0 | 0 | 0 | 0 | 0 | 0 | 1 | 1 | 2 | 4 | 0 | 0 | 0 | 0 | 1 | 0 | 0 | 0 | 0 | 0 | 0 | 0 |
| *Dendritina striata* | 0 | 9 | 0 | 0 | 0 | 0 | 0 | 0 | 0 | 0 | 0 | 0 | 0 | 0 | 1 | 0 | 2 | 4 | 0 | 0 | 0 | 0 | 0 | 0 | 0 | 0 | 0 | 0 | 0 | 0 |
| *Elphidium advenum* | 4 | 9 | 1 | 0 | 0 | 0 | 2 | 2 | 0 | 0 | 0 | 0 | 0 | 0 | 1 | 0 | 0 | 0 | 0 | 0 | 0 | 0 | 0 | 0 | 0 | 0 | 1 | 2 | 0 | 1 |
| *Elphidium crispum* | 0 | 9 | 5 | 0 | 1 | 0 | 3 | 9 | 4 | 1 | 2 | 2 | 1 | 0 | 6 | 8 | 7 | 4 | 2 | 0 | 0 | 4 | 0 | 0 | 5 | 3 | 6 | 5 | 2 | 3 |
| *Elphidium neosimplex* | 3 | 9 | 2 | 0 | 0 | 0 | 0 | 0 | 0 | 0 | 0 | 0 | 0 | 0 | 0 | 0 | 1 | 0 | 0 | 0 | 0 | 0 | 0 | 0 | 0 | 0 | 0 | 0 | 0 | 0 |
| *Eponides cribrorepandus* | 2 | 9 | 1 | 0 | 0 | 0 | 0 | 0 | 0 | 4 | 2 | 0 | 0 | 3 | 0 | 0 | 0 | 0 | 0 | 0 | 0 | 0 | 1 | 2 | 1 | 0 | 0 | 0 | 1 | 0 |
| *Eponides repandus* | 1 | 9 | 3 | 0 | 0 | 0 | 1 | 0 | 0 | 3 | 5 | 1 | 0 | 2 | 0 | 0 | 0 | 0 | 0 | 0 | 0 | 1 | 3 | 1 | 0 | 1 | 1 | 4 | 0 | 2 |
| *Fijella simplex* | 1 | 9 | 0 | 0 | 0 | 0 | 0 | 0 | 0 | 0 | 0 | 0 | 0 | 1 | 4 | 0 | 0 | 0 | 0 | 0 | 0 | 0 | 0 | 1 | 0 | 0 | 0 | 0 | 0 | 0 |
| *Hesterostegina depressa* | 0 | 9 | 1 | 4 | 1 | 5 | 0 | 0 | 0 | 0 | 3 | 3 | 5 | 5 | 0 | 0 | 0 | 0 | 1 | 0 | 0 | 1 | 1 | 0 | 1 | 0 | 0 | 0 | 1 | 0 |
| *Heterolepa dutemplei* | 0 | 9 | 0 | 0 | 0 | 0 | 0 | 0 | 1 | 0 | 0 | 0 | 0 | 0 | 0 | 0 | 0 | 0 | 0 | 0 | 0 | 0 | 0 | 2 | 1 | 3 | 0 | 0 | 0 | 0 |
| *Heterolepa subhaidingerii* | 0 | 9 | 0 | 0 | 0 | 0 | 0 | 0 | 0 | 0 | 0 | 0 | 0 | 0 | 1 | 3 | 0 | 0 | 0 | 0 | 0 | 0 | 0 | 0 | 0 | 0 | 0 | 0 | 0 | 0 |
| *Lachlanella compressiostoma* | 1 | 9 | 1 | 0 | 0 | 2 | 1 | 1 | 0 | 0 | 1 | 0 | 2 | 1 | 4 | 0 | 12 | 1 | 7 | 3 | 0 | 0 | 1 | 0 | 1 | 0 | 0 | 1 | 0 | 2 |
| *Mikrobelodontos bradyi* | 0 | 9 | 0 | 0 | 0 | 0 | 0 | 0 | 0 | 0 | 0 | 0 | 1 | 1 | 0 | 0 | 1 | 0 | 3 | 1 | 0 | 2 | 0 | 0 | 0 | 0 | 1 | 0 | 0 | 1 |
| *Millettiana milletti* | 0 | 9 | 0 | 0 | 0 | 0 | 0 | 0 | 0 | 1 | 0 | 0 | 0 | 0 | 5 | 0 | 0 | 0 | 1 | 0 | 0 | 0 | 0 | 0 | 1 | 0 | 0 | 0 | 0 | 0 |
| *Milliolinella suborbicularis* | 0 | 9 | 0 | 0 | 0 | 0 | 0 | 0 | 0 | 1 | 0 | 0 | 1 | 3 | 0 | 3 | 0 | 0 | 2 | 2 | 0 | 0 | 0 | 0 | 0 | 0 | 0 | 0 | 0 | 1 |
| *Nonionoides grateloupii* | 0 | 9 | 0 | 0 | 0 | 0 | 0 | 0 | 0 | 0 | 0 | 0 | 0 | 0 | 4 | 0 | 0 | 0 | 0 | 0 | 0 | 0 | 0 | 0 | 0 | 0 | 0 | 0 | 0 | 0 |
| *Nummulites venosus* | 4 | 9 | 2 | 3 | 1 | 3 | 1 | 1 | 0 | 0 | 2 | 2 | 0 | 0 | 0 | 0 | 3 | 28 | 0 | 0 | 34 | 1 | 0 | 0 | 9 | 4 | 8 | 1 | 4 | 7 |
| *Operculina discoidalis* | 0 | 9 | 0 | 0 | 0 | 0 | 0 | 0 | 0 | 0 | 0 | 0 | 0 | 0 | 0 | 0 | 0 | 0 | 0 | 0 | 3 | 0 | 0 | 0 | 0 | 0 | 0 | 0 | 0 | 0 |
| *Parahourinoides fragillissimus* | 0 | 9 | 0 | 0 | 0 | 0 | 0 | 0 | 0 | 0 | 0 | 3 | 0 | 0 | 0 | 0 | 0 | 0 | 0 | 0 | 0 | 0 | 0 | 0 | 0 | 1 | 0 | 0 | 0 | 0 |
| *Pararotalia calcariformata* | 0 | 9 | 0 | 0 | 0 | 0 | 0 | 0 | 0 | 0 | 0 | 0 | 0 | 0 | 0 | 4 | 0 | 0 | 1 | 0 | 0 | 0 | 0 | 0 | 4 | 0 | 0 | 0 | 0 | 0 |
| *Pararotalia domantayi* | 8 | 9 | 6 | 2 | 0 | 0 | 10 | 0 | 11 | 0 | 0 | 0 | 8 | 2 | 0 | 0 | 1 | 0 | 1 | 0 | 0 | 2 | 4 | 1 | 15 | 3 | 3 | 6 | 4 | 2 |
| *Parrelina hispidulla* | 0 | 9 | 0 | 0 | 0 | 0 | 0 | 0 | 0 | 0 | 0 | 0 | 0 | 1 | 6 | 1 | 2 | 6 | 1 | 0 | 0 | 0 | 0 | 0 | 0 | 1 | 1 | 1 | 1 | 1 |
| *Peneroplis pertusus* | 1 | 9 | 2 | 0 | 1 | 1 | 5 | 7 | 3 | 0 | 7 | 10 | 4 | 4 | 1 | 5 | 0 | 1 | 6 | 4 | 1 | 7 | 2 | 2 | 1 | 5 | 6 | 2 | 0 | 3 |
| *Peneroplis planatus* | 2 | 9 | 0 | 0 | 0 | 0 | 1 | 1 | 0 | 0 | 1 | 8 | 1 | 3 | 0 | 0 | 1 | 0 | 0 | 2 | 0 | 1 | 0 | 0 | 0 | 0 | 0 | 0 | 0 | 1 |
| *Pseudorotalia indopacifica* | 0 | 9 | 0 | 0 | 0 | 0 | 0 | 0 | 0 | 0 | 0 | 0 | 0 | 0 | 0 | 0 | 0 | 0 | 0 | 0 | 0 | 4 | 0 | 0 | 0 | 0 | 2 | 0 | 0 | 0 |
| *Quinqueloculina cuvieriana* | 0 | 9 | 0 | 0 | 0 | 0 | 1 | 0 | 0 | 1 | 1 | 1 | 1 | 0 | 3 | 8 | 0 | 0 | 0 | 1 | 0 | 1 | 1 | 1 | 5 | 3 | 2 | 1 | 0 | 1 |
| *Quinqueloculina incisa* | 0 | 9 | 0 | 0 | 0 | 0 | 0 | 0 | 0 | 0 | 0 | 0 | 0 | 0 | 0 | 2 | 1 | 0 | 0 | 0 | 0 | 3 | 1 | 0 | 0 | 0 | 0 | 1 | 0 | 0 |
| *Quinqueloculina parvaggluta* | 1 | 9 | 1 | 0 | 0 | 0 | 0 | 0 | 0 | 0 | 0 | 0 | 0 | 0 | 1 | 0 | 0 | 0 | 0 | 4 | 5 | 0 | 0 | 0 | 1 | 6 | 0 | 0 | 0 | 0 |
| *Quinqueloculina philippinensis* | 0 | 9 | 0 | 0 | 0 | 0 | 0 | 0 | 0 | 0 | 0 | 0 | 0 | 0 | 3 | 0 | 2 | 5 | 0 | 0 | 1 | 0 | 0 | 0 | 1 | 0 | 0 | 0 | 0 | 2 |
| *Quinqueloculina sulcata* | 0 | 9 | 0 | 1 | 0 | 0 | 0 | 0 | 0 | 0 | 0 | 0 | 1 | 1 | 0 | 0 | 0 | 0 | 0 | 0 | 0 | 3 | 1 | 0 | 0 | 0 | 0 | 0 | 0 | 0 |
| *Quinqueloculina vandiemeniensis* | 0 | 9 | 0 | 0 | 0 | 0 | 0 | 0 | 0 | 0 | 0 | 0 | 0 | 0 | 10 | 2 | 0 | 0 | 1 | 1 | 0 | 0 | 0 | 0 | 0 | 0 | 0 | 0 | 0 | 0 |
| *Rosalina globularis* | 0 | 9 | 0 | 0 | 0 | 0 | 0 | 0 | 0 | 4 | 0 | 0 | 0 | 0 | 0 | 1 | 1 | 0 | 0 | 0 | 0 | 0 | 0 | 0 | 0 | 1 | 0 | 1 | 0 | 0 |
| *Textularia agglutinans* | 0 | 9 | 0 | 0 | 1 | 0 | 0 | 0 | 1 | 0 | 1 | 1 | 0 | 0 | 0 | 0 | 0 | 0 | 4 | 0 | 0 | 0 | 0 | 0 | 0 | 0 | 0 | 0 | 0 | 0 |
| *Textularia lateralis* | 0 | 9 | 0 | 0 | 1 | 0 | 0 | 0 | 2 | 6 | 0 | 2 | 0 | 0 | 0 | 0 | 0 | 0 | 0 | 6 | 0 | 1 | 0 | 0 | 1 | 0 | 0 | 0 | 0 | 0 |
| *Triloculina marshallana* | 0 | 9 | 2 | 0 | 0 | 1 | 0 | 1 | 0 | 0 | 0 | 0 | 0 | 0 | 0 | 0 | 0 | 0 | 1 | 6 | 2 | 0 | 0 | 0 | 0 | 1 | 1 | 1 | 0 | 3 |
| *Triloculina tricarinata* | 0 | 9 | 0 | 0 | 0 | 0 | 0 | 3 | 9 | 0 | 3 | 4 | 1 | 0 | 1 | 6 | 8 | 0 | 7 | 0 | 0 | 0 | 0 | 0 | 0 | 1 | 0 | 1 | 0 | 1 |
| *Triloculinella bertheliniana* | 0 | 9 | 0 | 0 | 1 | 0 | 3 | 0 | 0 | 0 | 0 | 0 | 0 | 0 | 0 | 0 | 0 | 0 | 0 | 0 | 1 | 0 | 0 | 0 | 1 | 0 | 0 | 0 | 0 | 0 |
| *Triloculinella parisa* | 0 | 9 | 0 | 0 | 0 | 0 | 0 | 0 | 0 | 0 | 0 | 0 | 0 | 0 | 0 | 0 | 0 | 0 | 3 | 0 | 0 | 1 | 0 | 0 | 0 | 0 | 0 | 0 | 0 | 1 |
| *Triloculinella chiastocytis* | 0 | 9 | 0 | 0 | 0 | 0 | 0 | 0 | 0 | 0 | 0 | 0 | 0 | 0 | 3 | 1 | 7 | 0 | 0 | 1 | 0 | 1 | 0 | 0 | 0 | 0 | 0 | 0 | 0 | 0 |
